# Supplementary material for: Molecular architecture and regulation of BCL10-MALT1 filaments
Source: Nat Commun. 2018 Oct 2;9:4041. doi: 10.1038/s41467-018-06573-8 (PMC6168461; doi:10.1038/s41467-018-06573-8)
Supplement: Supplementary file 1 — Supplementary Information [file 41467_2018_6573_MOESM1_ESM.pdf]

Supplementary Information for

Molecular architecture and regulation of BCL10-MALT1 filaments

Schlauderer et al.

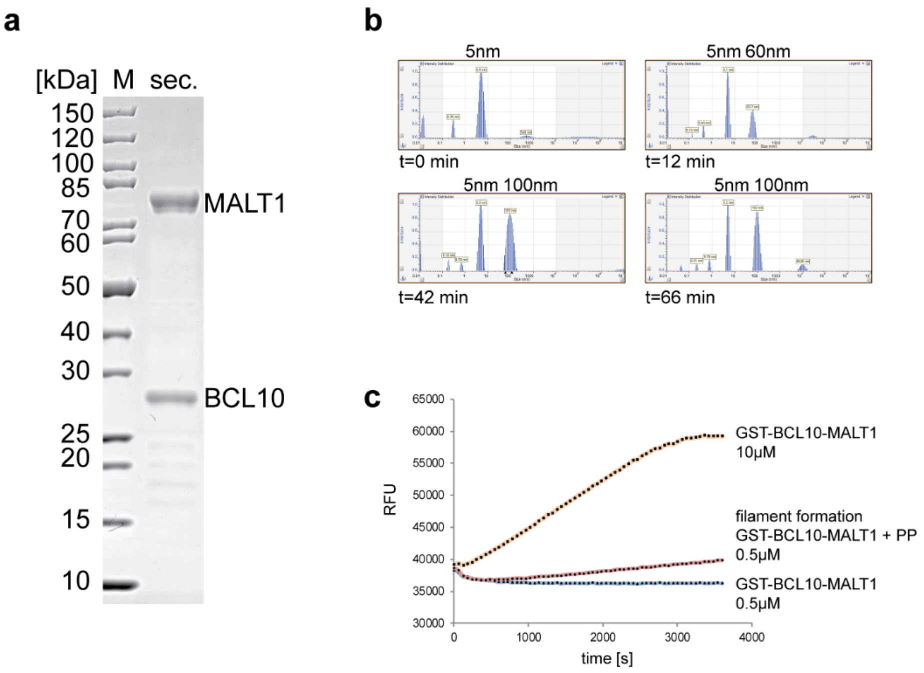

Supplementary Figure 1: Biochemical analysis of the recombinant BCL10-MALT1 complex. (a) SDS Page analysis of the purified BCL10-MALT1 protein complex used for cryo-EM grid preparation and biochemical analysis. (b) Dynamic light scattering (DLS) experiment showing the polymerization of the BCL10 MALT1 complex purified from *E. coli* after cleavage of the GST-tag by PreScission protease. c) Fluorescence activity measurement using the MALT1 substrate Ac-LRSR-AMC. The MALT1 protease activity is represented in relative fluorescence units (RFU).

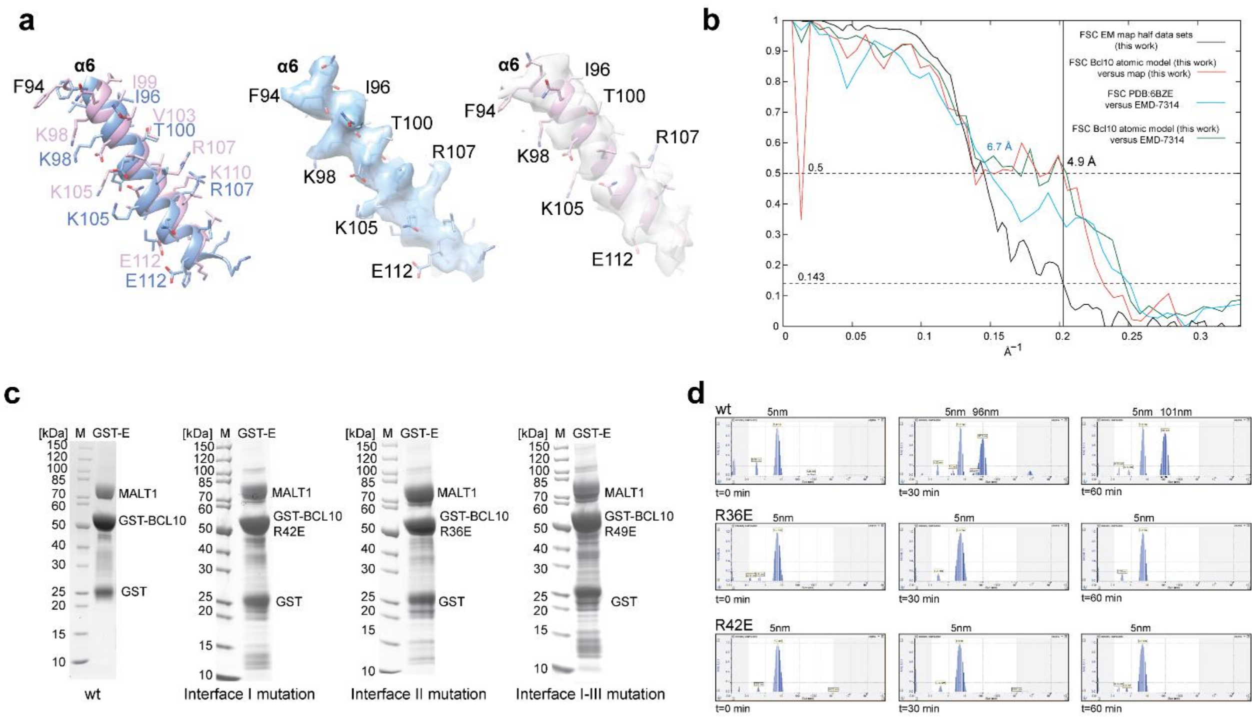

Supplementary Figure 2: Biochemical analysis of the recombinant BCL10-MALT1 complex wt and mutants. (a) Comparison of the BCL10 EM structures alone (colored pink) and inside the BCL10-MALT1 complex[31] (colored blue) indicates differences in the amino acid registry in helix α6 (left). Exemplary model versus map comparison of our BCL10 model and the BCL10 filament structure without MALT1[31](EMD entry: 7314) middle and right respectively. (b) FSC between refined model and final map. The resolution of 4.9 Å was determined using the 0.143 FSC criterion as indicated by the dotted line. (c) SDS PAGE analysis of GST affinity chromatography elution fractions of BCL10-MALT1 wt and BCL10 mutant proteins. (d) Oligomerization proficiency of wt, R42E and R36E BCL10 mutants monitored by DLS analysis of the respective recombinant BCL10-MALT1 complexes.

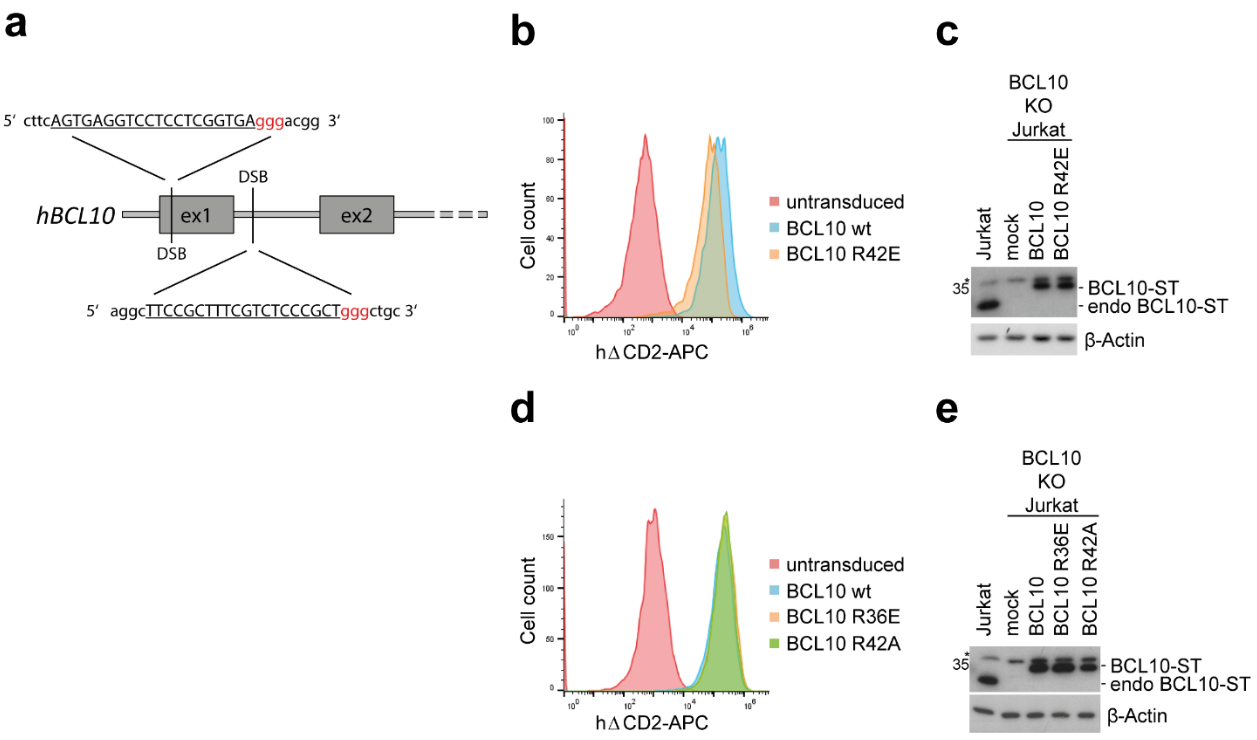

Supplementary Figure 3: Generation of BCL10 KO Jurkat T-cells and transduction efficiency of BCL10 constructs. (a) Scheme of genomic organization (ex1-ex2) of human BCL10 gene and depiction of sgRNAs used to delete parts of exon 1 (ex1). (b-e) Transduction efficiency of BCL10 constructs. Infection of BCL10 KO Jurkat T-cells was determined by the co-expressed surface marker hΔCD2 in FACS (b, d). Expression of BCL10 and BCL10 mutants in reconstituted BCL10 KO cells compared to parental Jurkat T-cells was analyzed in WB (c, e). Asterisk indicates non-specific cross-reactivity of BCL10 antibody.

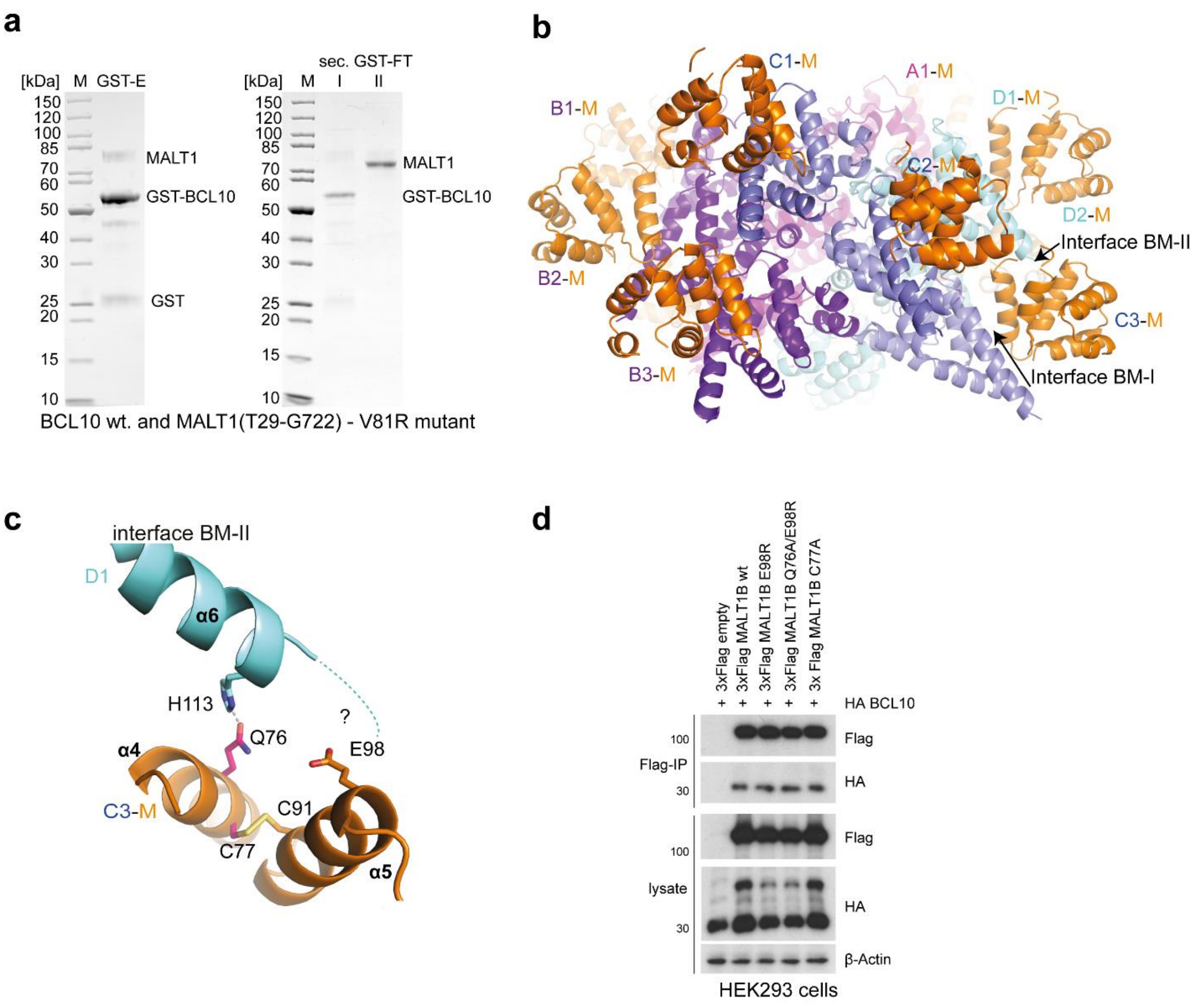

Supplementary Figure 4: Structural and functional analyses of BCL10-MALT1 interfaces. (a) SDS PAGE analysis of GST affinity chromatography elution fractions of BCL10 with MALT1 (T29-G722) or MALT1 (T29-G722) V81R. (b) Side view of one repeat of the BCL10-MALT1 holo-complex as visible in the cryo-EM density. Position of the putative interface BM-II is indicated. (c) Close up view of the putative BM-II shown as ribbon model in purple (BCL10) and orange (MALT1). Potentially interacting residues are shown in stick representation. Mutations introduced are colored magenta. (d) HEK293 cells were co-transfected with 3xFLAG MALT1B wt and indicated BM-II mutant constructs and HA BCL10 wt. Co-IP was carried out using anti-Flag antibodies and analyzed by WB.

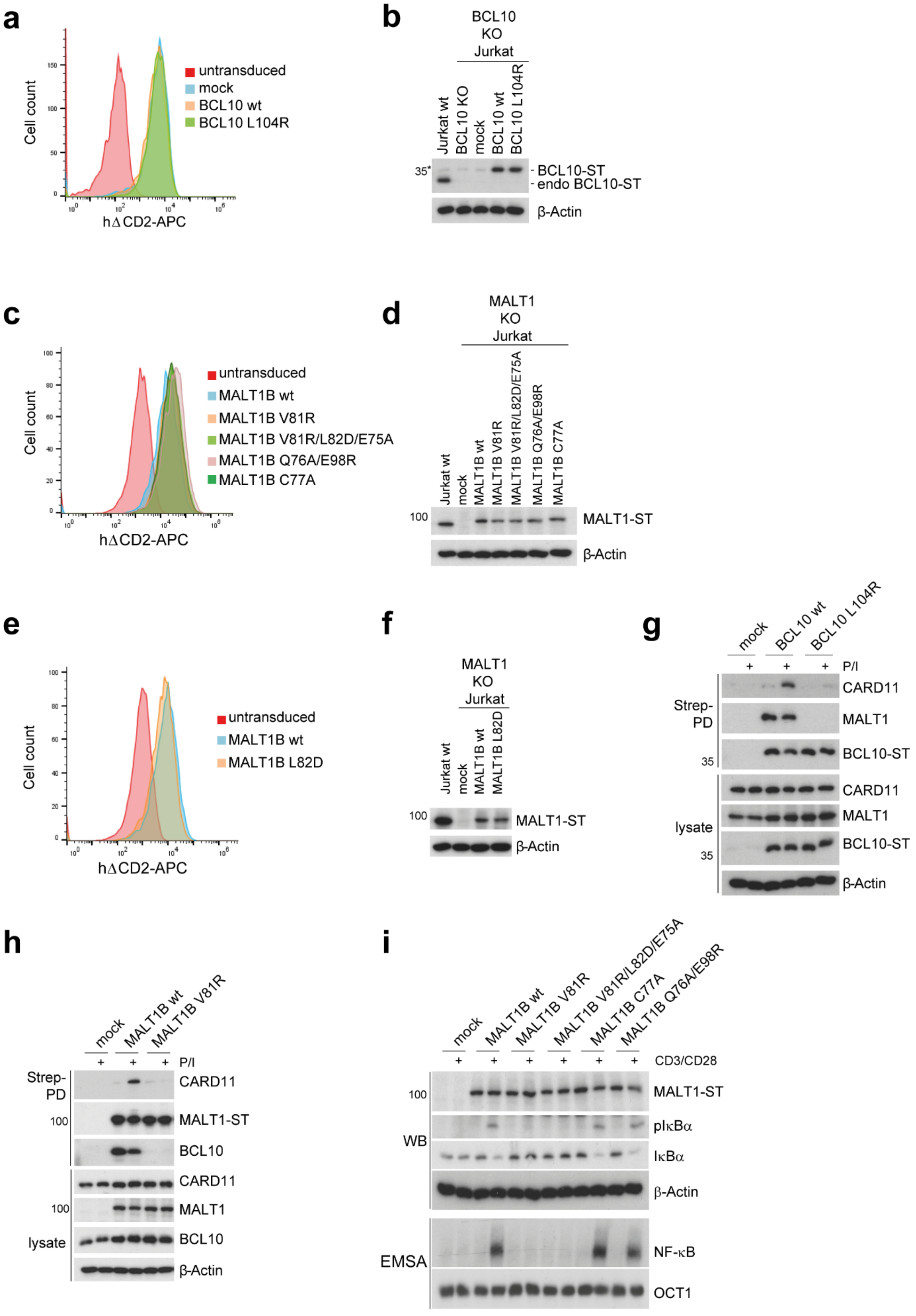

a
Cell count
hΔCD2-APC
untransduced
mock
BCL10 wt
BCL10 L104R
b
BCL10 KO Jurkat
Jurkat wt
BCL10 KO
mock
BCL10 wt
BCL10 L104R
35*
BCL10-ST
endo BCL10-ST
β-Actin
c
Cell count
hΔCD2-APC
untransduced
MALT1B wt
MALT1B V81R
MALT1B V81R/L82D/E75A
MALT1B Q76A/E98R
MALT1B C77A
d
MALT1 KO Jurkat
Jurkat wt
mock
MALT1B wt
MALT1B V81R
MALT1B V81R/L82D/E75A
MALT1B Q76A/E98R
MALT1B C77A
100
MALT1-ST
β-Actin
e
Cell count
hΔCD2-APC
untransduced
MALT1B wt
MALT1B L82D
f
MALT1 KO Jurkat
Jurkat wt
mock
MALT1B wt
MALT1B L82D
100
MALT1-ST
β-Actin
g
mock
BCL10 wt
BCL10 L104R
P/I
Strep-PD
CARD11
MALT1
35
BCL10-ST
lysate
CARD11
MALT1
35
BCL10-ST
β-Actin
h
mock
MALT1B wt
MALT1B V81R
P/I
Strep-PD
CARD11
100
MALT1-ST
BCL10
lysate
CARD11
100
MALT1
BCL10
β-Actin
i
mock
MALT1B wt
MALT1B V81R
MALT1B V81R/L82D/E75A
MALT1B C77A
MALT1B Q76A/E98R
CD3/CD28
WB
100
MALT1-ST
pIκBα
IκBα
β-Actin
EMSA
NF-κB
OCT1

Supplementary Figure 5: Transduction efficiency of BCL10 and MALT1 wt and BM-I mutants in Jurkat T-cells. (a) Transduction of BCL10 wt and mutant L104R in BCL10 KO Jurkat T-cells determined by the co-expressed surface marker h∆CD2 in FACS. (b) Expression of Strep-tagged BCL10 and BCL10 L104R in reconstituted BCL10 KO cells and comparison to parental Jurkat T-cells in WB. Asterisk indicates non-specific cross-reactivity of BCL10 antibody. (c-f) Transduction of MALT1 constructs in MALT1 KO Jurkat T-cells. Transduction efficiency was determined by the co-expressed surface marker h∆CD2 in FACS (c, e). Expression of MALT1 or MALT1 BM-I or BM-II mutant constructs and comparison to parental Jurkat T-cells in WB (d, f). (g) MALT1 KO Jurkat T-cells reconstituted with MALT1 wt or V81R mutant were stimulated for 20 min with P/I stimulation and CBM complex formation was investigated by Strep-PD. (h) BCL10 KO Jurkat T-cells were reconstituted with BCL10 wt or L104R mutant constructs and stimulation and Strep-PD was carried out as described in g. (i) MALT1 KO Jurkat T-cells transduced with MALT1B wt, BM-I or BM-II mutants were stimulated with anti-CD3/28 for 30 minutes. NF-κB signaling was analyzed by WB and EMSA.

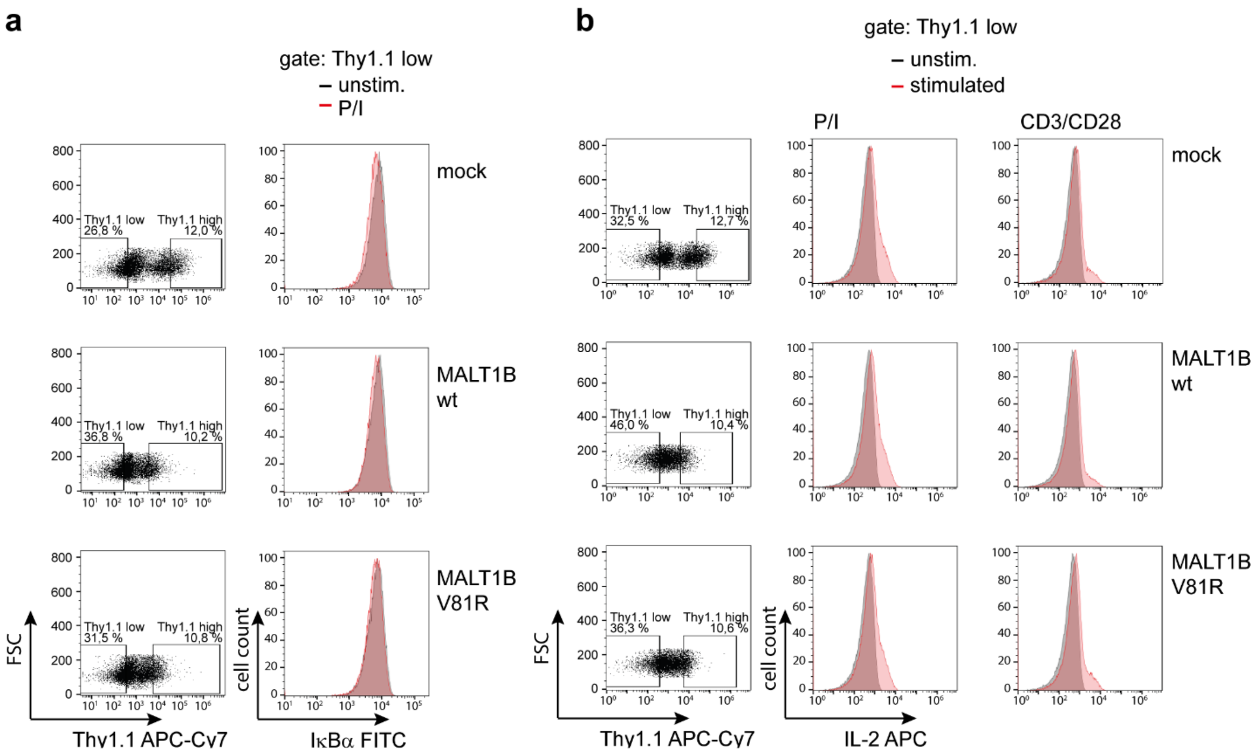

Supplementary Figure 6: Transduction of MALT1 wt and BM-I mutant V81R in murine MALT1$^{-/-}$ CD4 T-cells. (a Murine MALT1$^{-/-}$ CD4 T-cells were retrovirally transduced with mock, MALT1 wt or MALT1 BM-I mutants and the surface marker Thy1.1. For single cells analyses of NF-κB signaling, CD4 T-cells were untreated or stimulated with P/I (20 min) before staining with anti-IκBα (FITC) and Thy1.1-APC. IκBα expression was analyzed in Thy1.1-positive transduced cells or Thy1.1-negative untransduced cells in indicated gates. IκBα staining in the control untransduced Thy1.1-negative T-cell population (MALT1 deficient) is shown in the right histograms and IκBα expression levels in transduced Thy1.1-positive T-cells (mock or MALT1 rescue) is shown in Fig. 5j. (b) MALT1$^{-/-}$ CD4 T-cells were retrovirally transduced as in c. For single cells analyses of IL-2 production, CD4 T-cells were untreated or stimulated with P/I or anti-CD3/CD28 for 5 h before staining with anti-IL-2-APC and Thy1.1-APC-Cy7. Intracellular IL-2 production was analyzed in Thy1.1-positive transduced cells or Thy1.1-negative untransduced cells in indicated gates. IL-2 expression staining in the control untransduced Thy1.1-negative T-cell population (MALT1 deficient) is shown in the histograms and IL-2 expression levels in transduced Thy1.1-positive T-cells (mock or MALT1 rescue) are shown in Figure 5k (for P/I) and 5l (for anti-CD3/CD28).

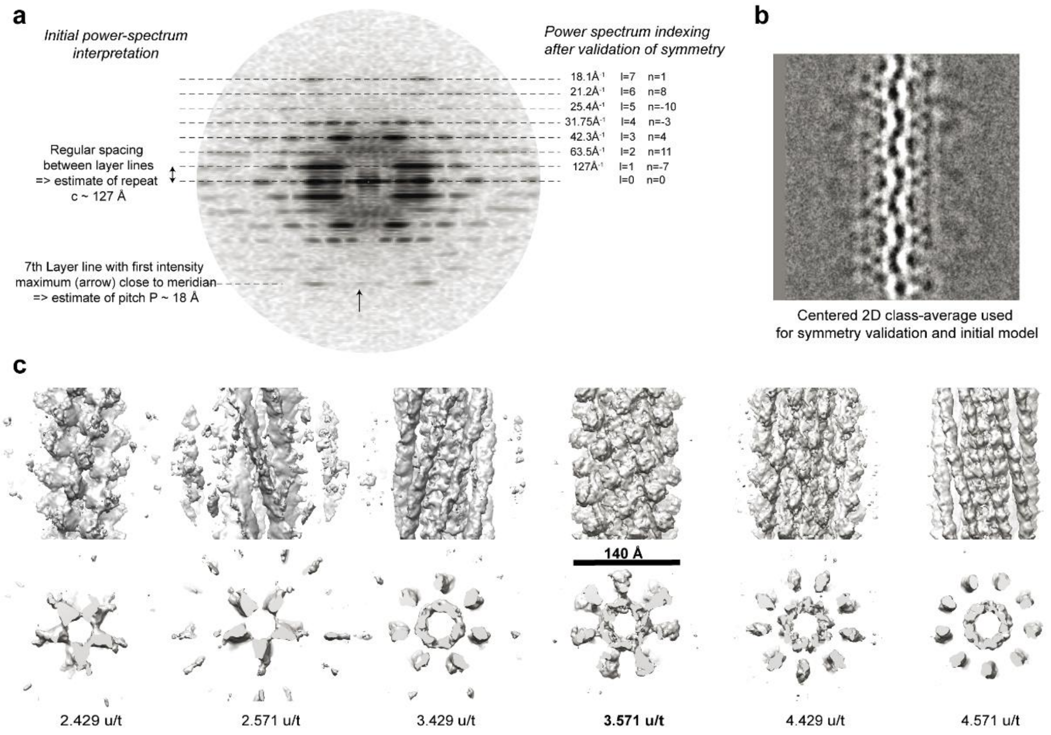

Supplementary Figure 7: Sum of the power spectra of selected 2D class-averages, its initial interpretation (left) and the indexing (right) corresponding to the final symmetry (pitch 18.15 Å, 3.571 units/turn) (b) The centered 2D class-average used by the module segclassreconstruct to explore plausible symmetries as determined from the initial analysis of the power spectrum shown in (a). (Scale bar = 100 Å). (c). 3D reconstructions obtained from the single 2D class-average shown in (b), for various symmetries for which the predicted layer line positions and their corresponding Bessel function order is compatible with the observed power spectrum. The correct symmetry is indicated in bold.

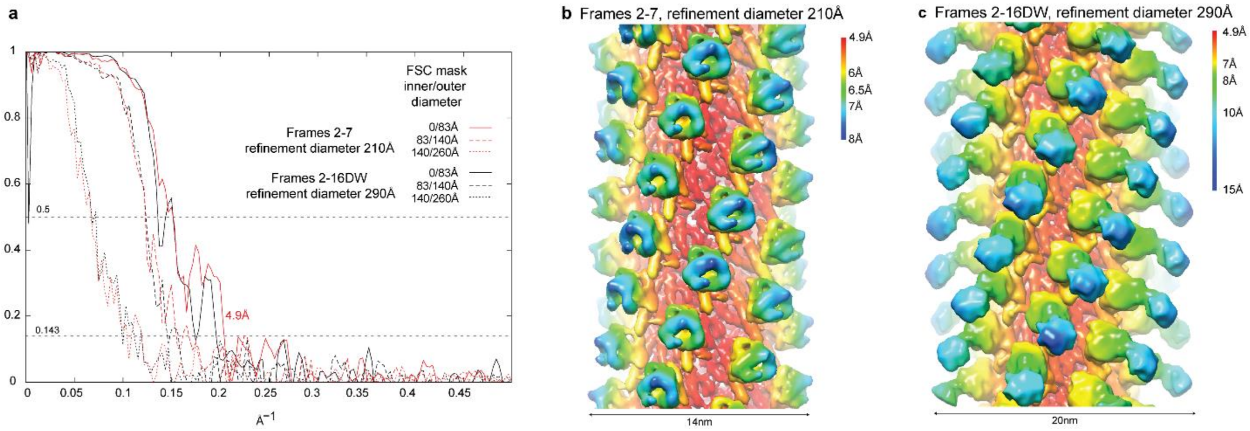

Supplementary Figure 8: (a) Fourier shell correlation (FSC) curves between half-maps from the two refinement procedures, with frames 2-7 (red curves) or 2-16 dose weighted (DW) (black curves). The FSCs were calculated within smooth cylinders of indicated inner and outer diameter. (b-c) Refined maps generated from the high (b) and low (c) resolution data evaluation procedure colored and filtered for local resolution using the indicated color scheme.

Figure 3a

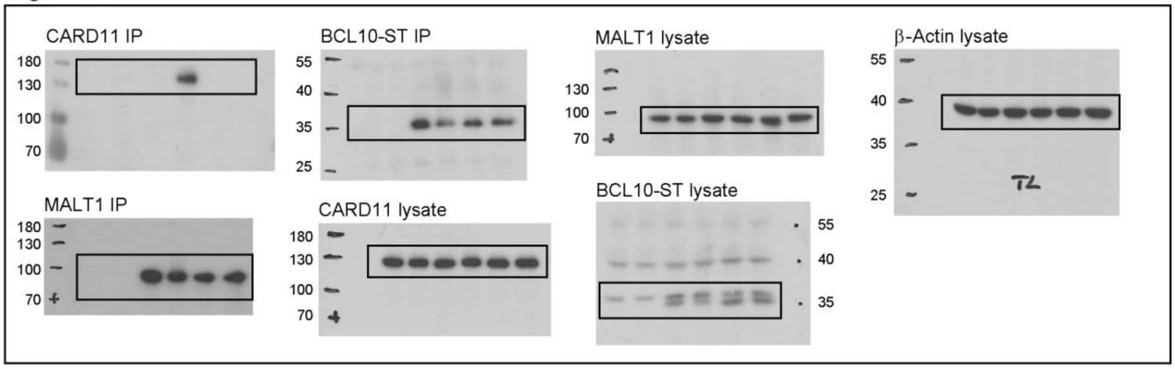

Figure 3b

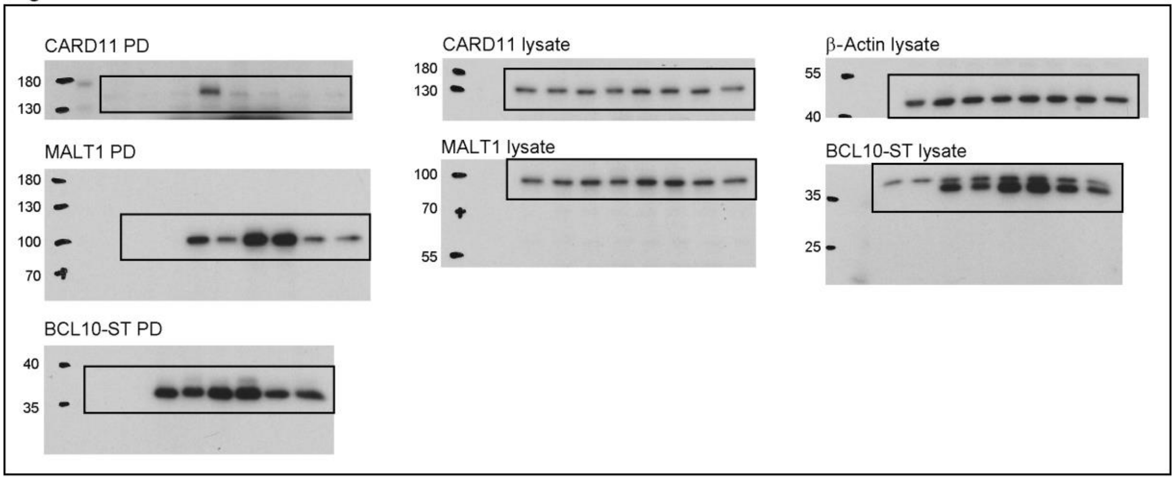

Figure 3c

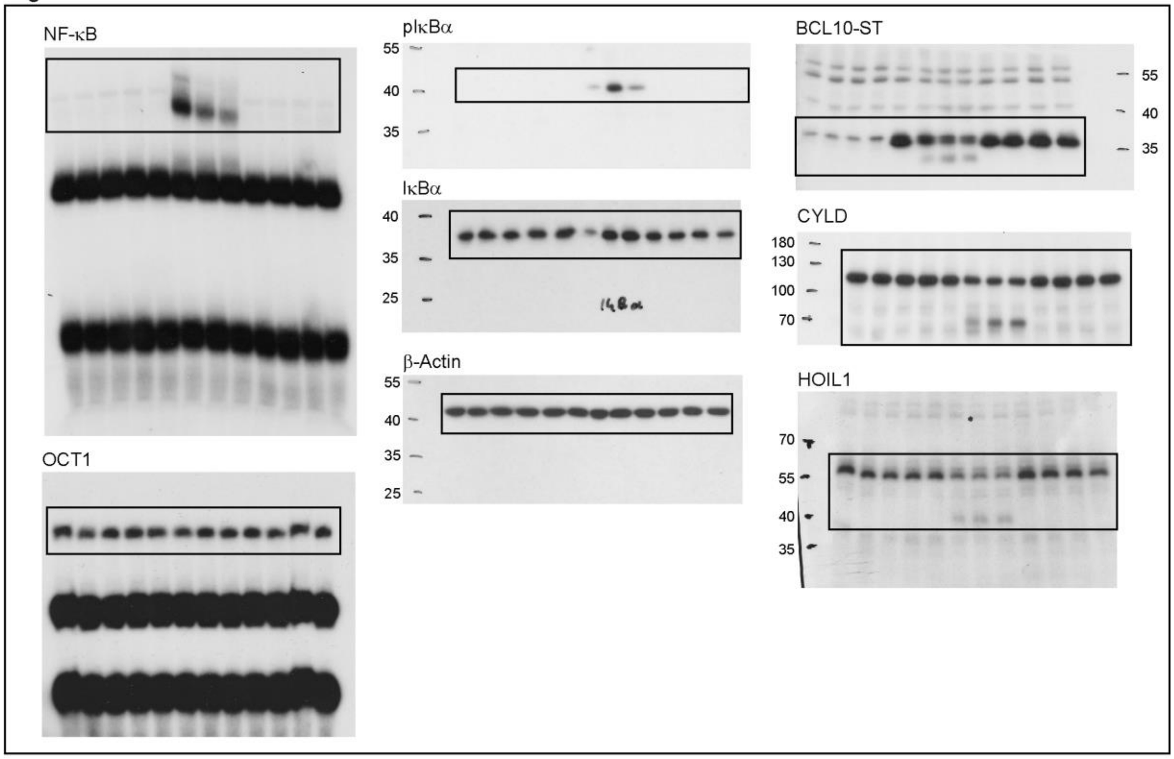

Supplementary Figure 9: Uncropped images. Boxes highlight approx. area for cropping.

Figure 3d

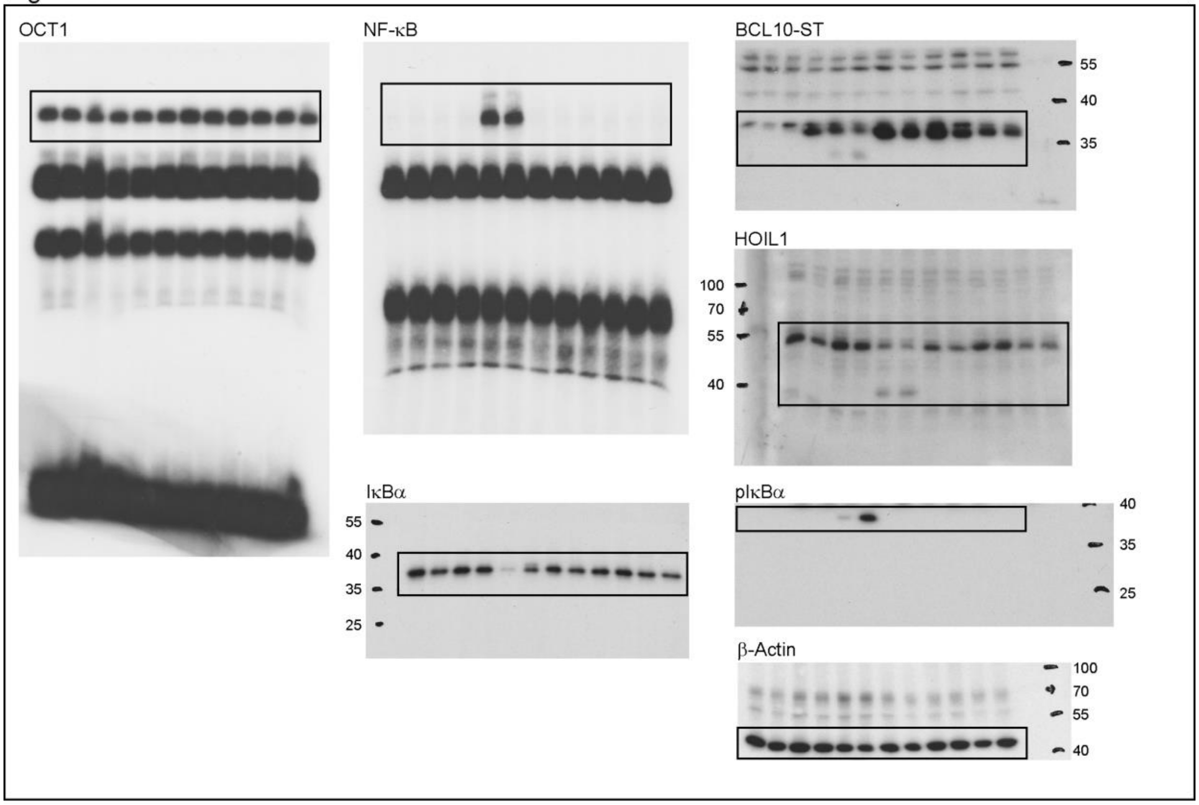

Figure 4d

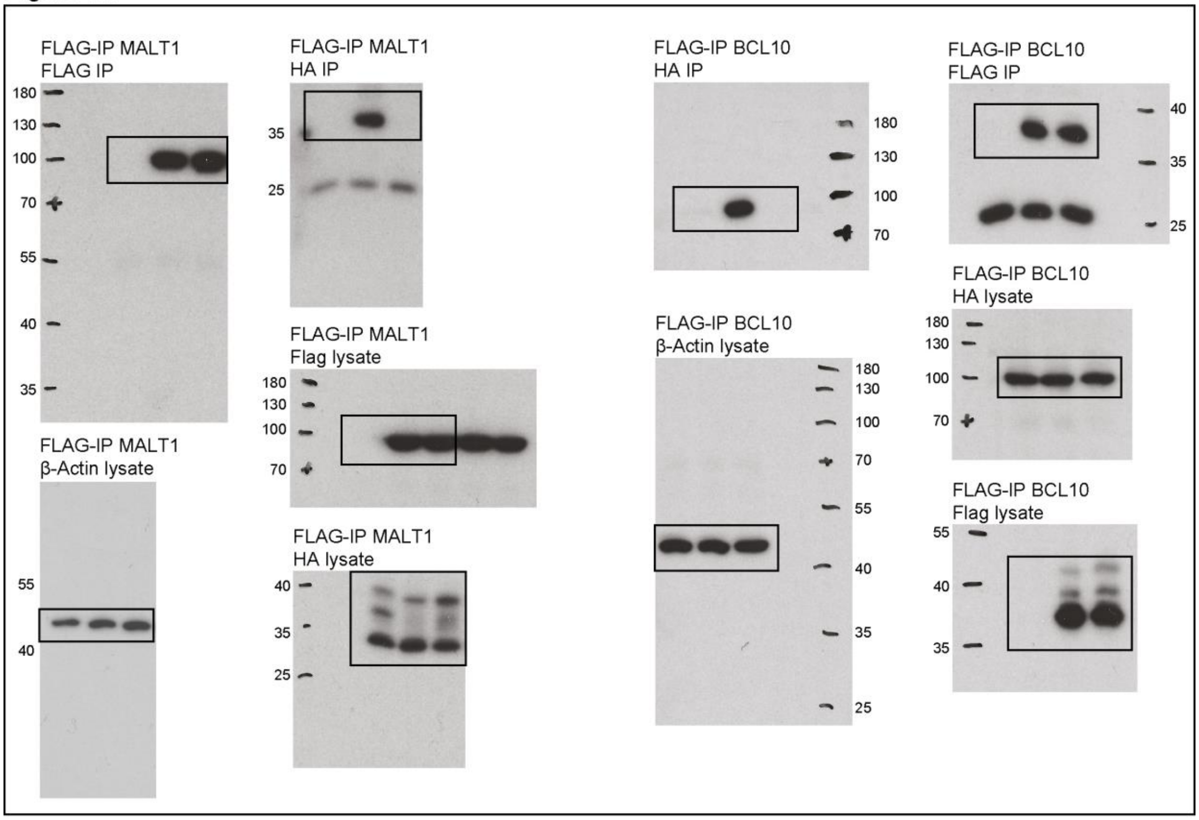

Supplementary Figure 9: Continued.

Figure 5a

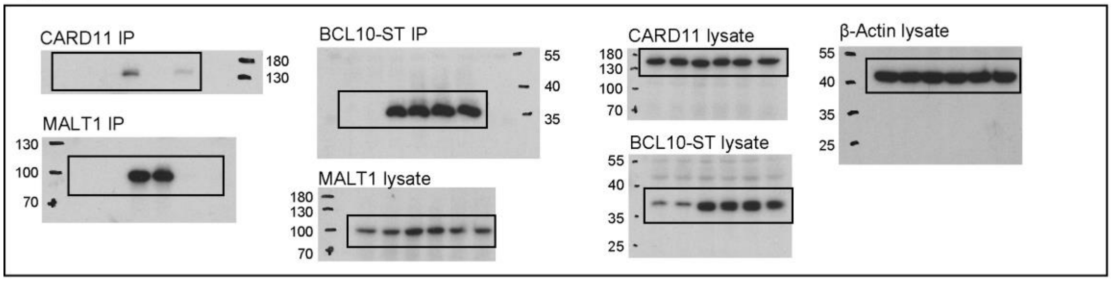

Figure 5b

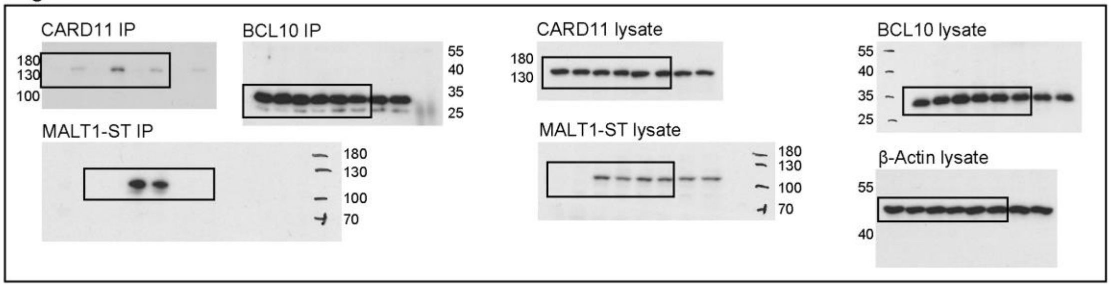

Figure 5c

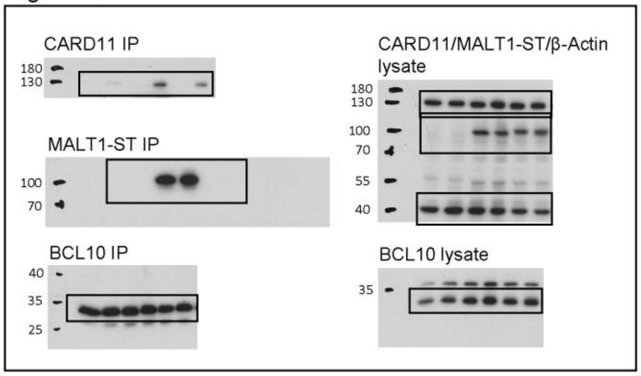

Figure 5d

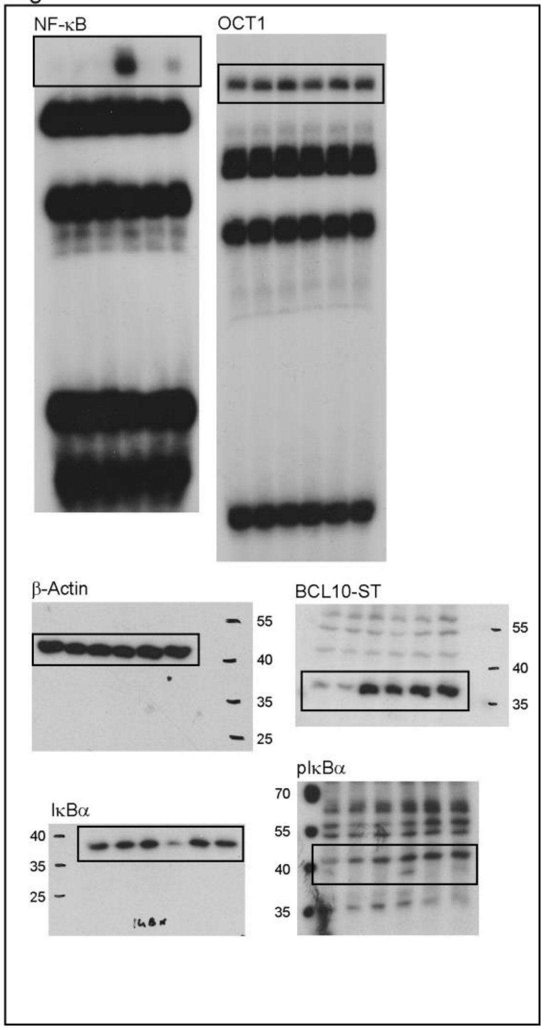

Figure 5e

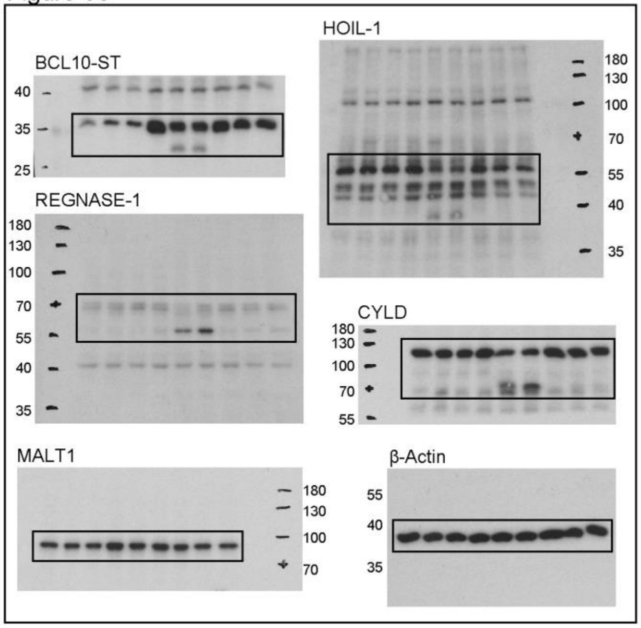

Supplementary Figure 9: Continued.

Figure 5f

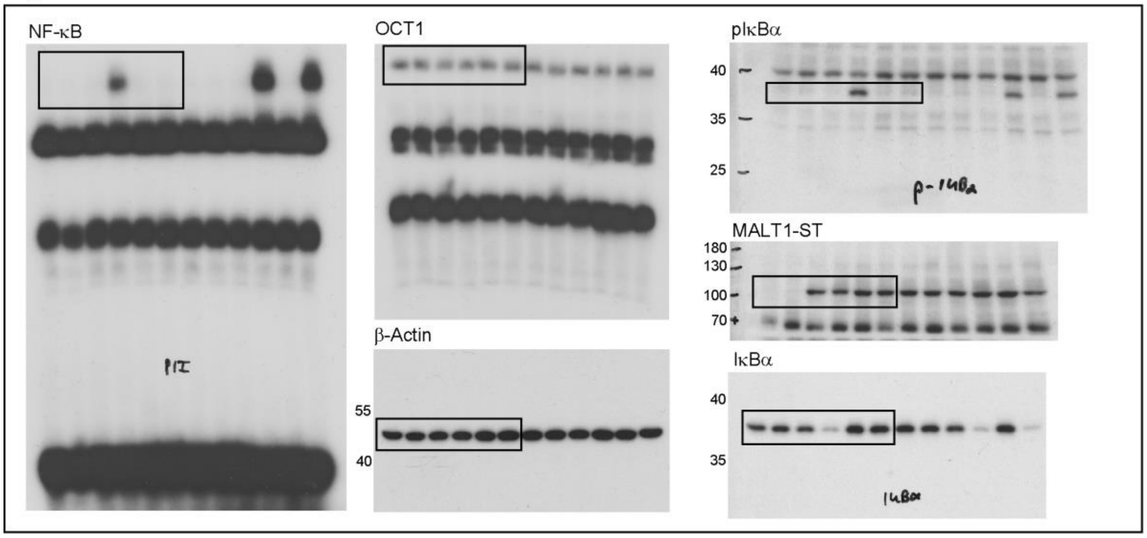

Figure 5g

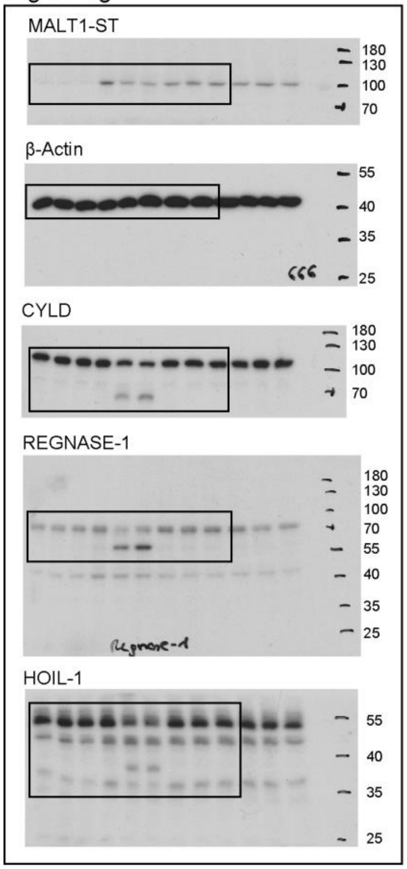

Figure 5h

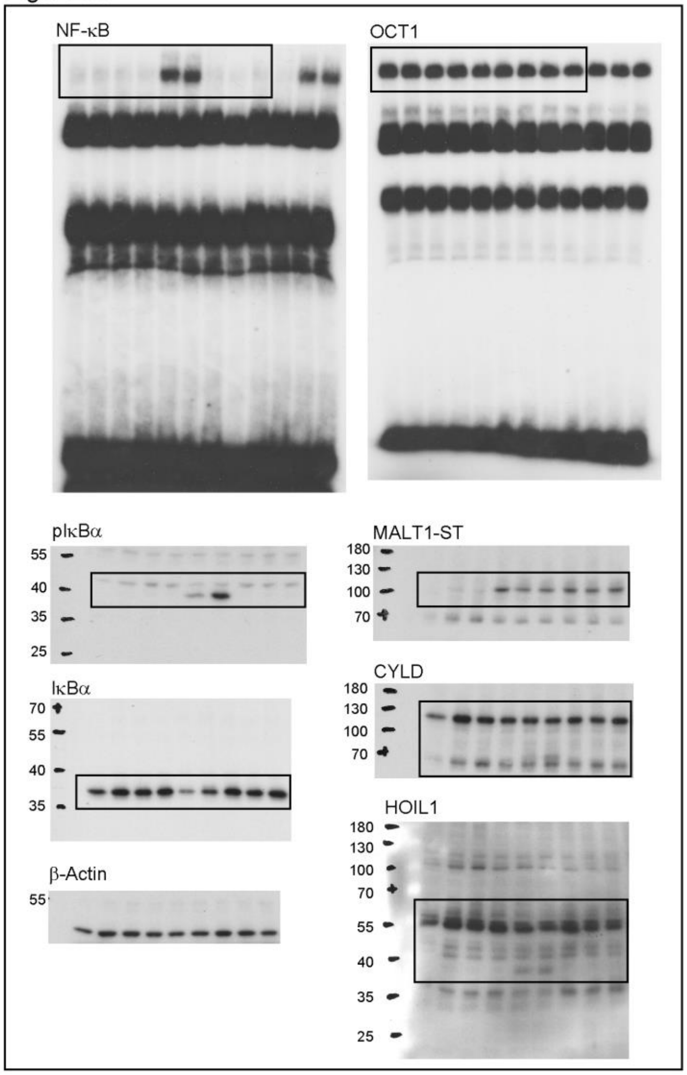

Figure 5i

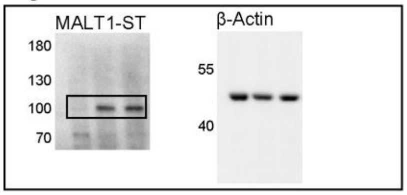

Supplementary Figure 9: Continued.

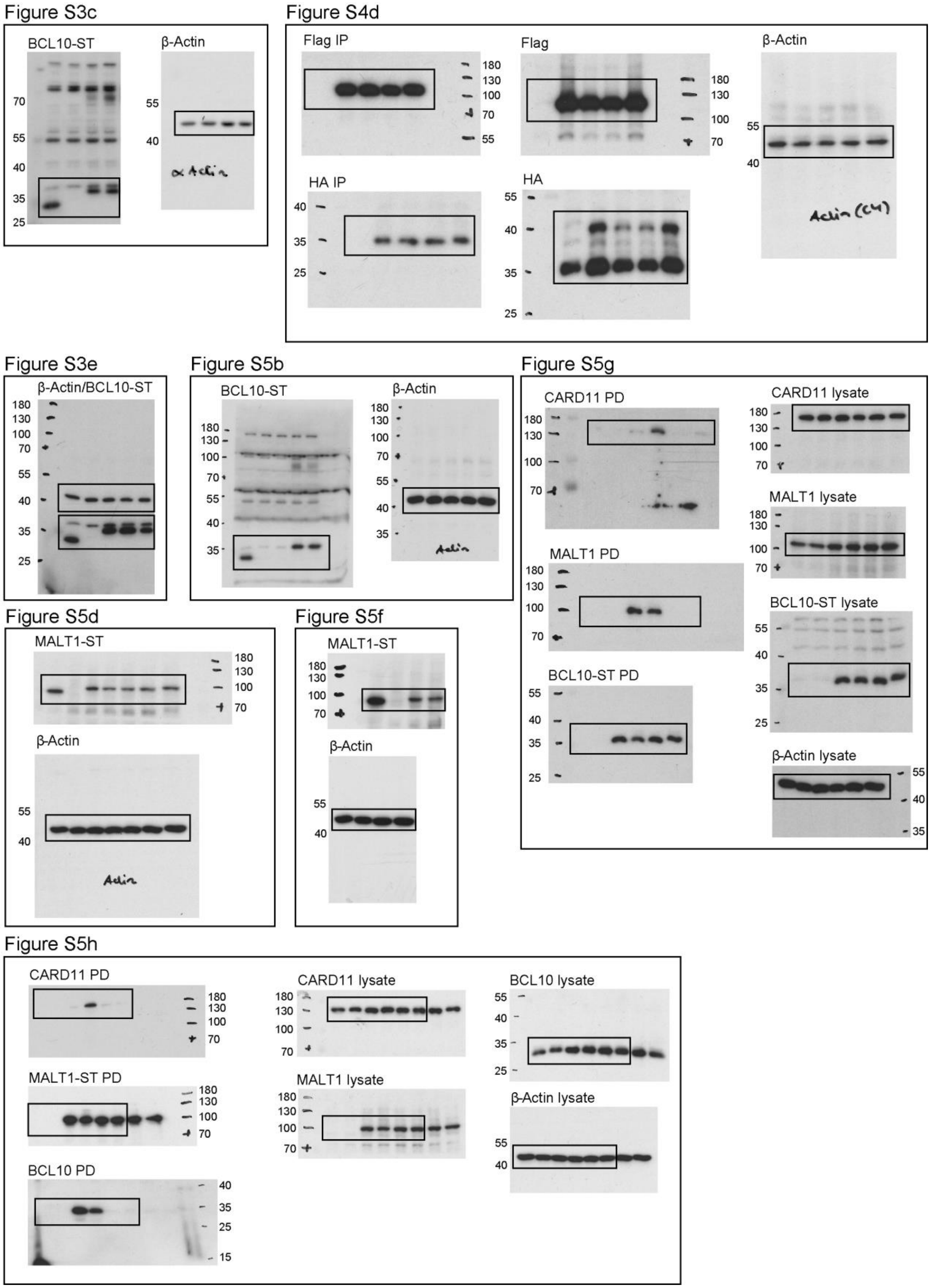

Supplementary Figure 9: Continued.

Figure S5i

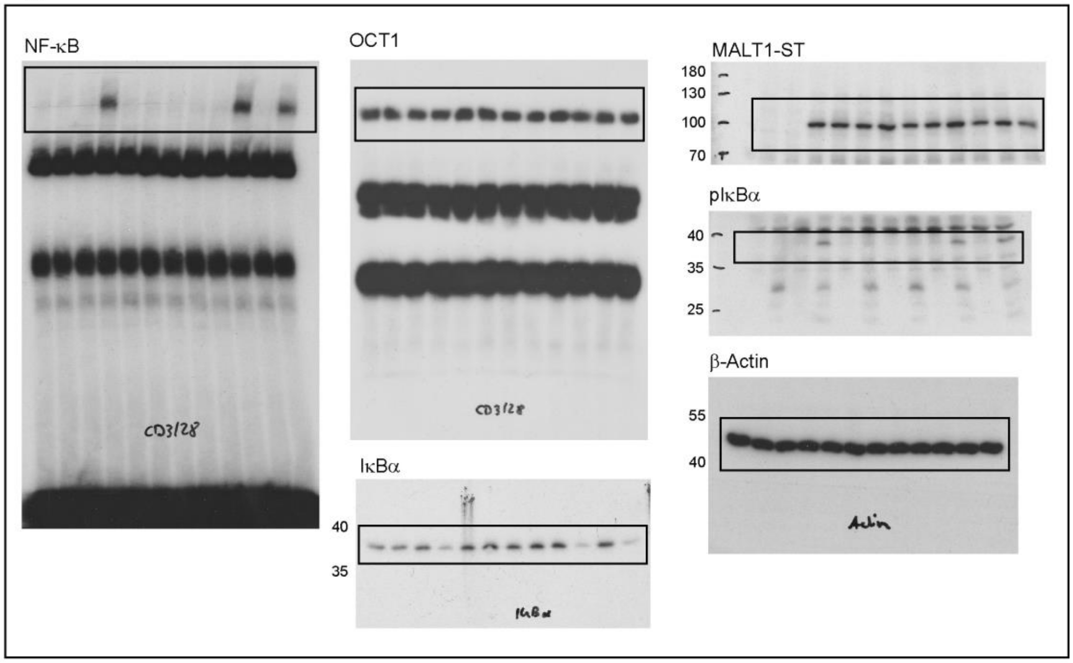

Supplementary Figure 9: Continued.

Supplementary Table 1: Cryo-EM data collection, refinement and validation statistics

**Data collection**	
Voltage (kV)	200
Electron Dose (e-Å^2)	99.6
Number of Frames	49
Detector	Falcon III
Pixel Size (Å)	1.002
Defocus range (-µm)	1.0-3.5
Total number of micrographs	370
Total number of filaments	2618
**Reconstruction high resolution**	
Electron Dose (e-Å^2)	15
Segment size (Å):	500
Segmentation step size (Å):	30
Initial segments:	25576
Final segments:	8591
Diameter (Å):	210
Helical rise (Å):	5.083
Helical twist (°):	-100.8
Map sharpening B factor (Å^2):	-200
Final resolution (Å):	6.9/4.9
FSC threshold:	0.5/0.143
**Reconstruction low resolution**	
Electron Dose (e-Å2)	32
Segment size (Å):	500
Segmentation step size (Å):	30
Initial segments:	25576
Final segments:	8412
Diameter (Å):	290
Helical rise (Å):	5.083

Helical twist (°):	-100.8
Final resolution (Å):	7.7/5.9
FSC threshold:	0.5/0.143

Refinement:

Initial models used (PDB code)	2G7R and 2MB9
Model resolution (Å)	4.9
FSC threshold	0.143
Model resolution range (Å)	30-4.9
Map sharpening B factor ($Å^2$)	-200
CC Model vs. Map	0.79
Model composition	
Non-hydrogen atoms	196
Protein residues	196
R.m.s. deviations	
Bond lengths (Å)	0.008
Bond angles (°)	0.93
Validation	
MolProbity score	-
Clashscore	
Poor rotamers (%)	0.1*
Ramachandran plot	
Favored (%)	92.8*
Allowed (%)	4.2*
Disallowed (%)	0.0*

*These values are not independent quality criteria, since, during refinement, restraints for rotamers and Ramachandran plot were used

Supplementary Table 2: Primer list

Construct	Forward primer	Reverse primer
MALT1 (T29-G722)	5‘-CCCTGGGCAGCCAT ATGACCCTCAACCGCCTG-3‘	5‘-TGCTCGAGTGCGGCC GCTCATCCCAAACCTCGATGCA-3‘
MALT1(V81R)	5‘-CAGTGTTCTCTTAAG CGACTGGAGCCTGAAG-3‘	5‘-CTTCAGGCTCCAGTC GCTTAAGAGAACACTG-3‘
BCL10 (L104R)	5‘-ACAGATGAAGTG CGGAAACTTAGAA-3’	5‘-TTCTAAGTTTC CGCACTTCATCTGT-3‘
BCL10 (R36E)	5‘-TCATAGCTGAGGAA CATTTTGATCATCTACGTG-3‘	5‘-CACGTAGATGATCAAA ATGTTCCTCAGCTATGA-3‘
BCL10 (R42E)	5‘-TTTGATCATCTAGAA GCAAAAAAAATACTCAG-3‘	5‘-CTGAGTATTTTTTTTG CTTCTAGATGATCAAA-3‘
BCL10 (R49E)	5‘- CGTGCAAAAAAAAT ACTCAGTGAAGAAGACACT-3‘	5‘-GTGTCTTCTTCACTGA GTATTTTTTTTGCACG-3‘

Supplementary Table 3: Oligonucleotide primer for generation of mutations

Mutation	Mutagenesis primer
MALT1 V81R	5‘-CAGTGTTCTCTTAAGCGACTGGAGCCTGAAG-3‘
MALT1 L82D	5‘-TTCAGGCTCGTCTACCTTAAGAGA-3‘
MALT1 E75A/V81R/L82D	5‘-AGGCTCATCTCTCTTAAGAGAACACTGCGCCAGG-3‘
MALT1 E98R	5‘-ACTGTGCAACCTTTTCTACCCATTAACTTCAG-3‘
MALT1 Q76A	5’-AGAGAACACGCCTCCAGGTCTAGGC-3’
MALT1 C77A	5‘-TACCTTAAGAGAAGCCTGCTCCAGGTCTA-3‘
BCL10 R36E	5‘-TCATAGCTGAGGAACATTTTGATCATCTACGTG-3‘
BCL10 R42E	5‘-TTTGATCATCTAGAAGCAAAAAAAATACTCAG-3‘
BCL10 R49E	5‘-CGTGCAAAAAAAATACTCAGTGAAGAAGACACT-3‘
BCL10 R42A	5‘-TTTTTGCAGCTAGATGATCA-3’
BCL10 L104R	5’-TAAGTTTCCGCACTTCATCTGTAAT-3’
